# Cost-effectiveness of implementing a suicide prediction tool (OxMIS) in severe mental illness: Economic modeling study

**DOI:** 10.1192/j.eurpsy.2022.2354

**Published:** 2022-12-19

**Authors:** Stella Botchway, Apostolos Tsiachristas, Jack Pollard, Seena Fazel

**Affiliations:** 1 Department of Psychiatry, University of Oxford, Oxford, United Kingdom; 2 Health Economics Research Centre, Nuffield Department of Population Health, University of Oxford, Oxford, United Kingdom

**Keywords:** risk prediction, suicide, prevention, mental health, cost-effectiveness

## Abstract

**Background:**

Cost-effectiveness analysis needs to be considered when introducing new tools and treatments to clinical services. The number of new assessment tools in mental health has rapidly expanded, including suicide risk assessment. Such suicide-based assessments, when linked to preventative interventions, are integral to high-quality mental health care for people with severe mental illness (SMI). We examined the cost implications of implementing Oxford Mental Illness and Suicide (OxMIS), an evidence-based, scalable suicide risk assessment tool that provides probabilistic estimates of suicide risk over 12 months for people with SMI in England.

**Methods:**

We developed a decision analytic model using secondary data to estimate the potential cost-effectiveness of incorporating OxMIS into clinical decision-making in secondary care as compared to usual care. Cost-effectiveness was measured in terms of costs per quality-adjusted life years (QALYs) gained. Uncertainty was addressed with deterministic and probabilistic sensitivity analysis.

**Results:**

Conducting suicide risk assessment with OxMIS was potentially cheaper than clinical risk assessment alone by £250 (95% confidence interval, −786;31) to £599 (−1,321;−156) (in 2020–2021 prices) per person with SMI and associated with a small increase in quality of life (0.01 [−0.03;0.05] to 0.01 QALY, [−0.04;0.07]). The estimated incremental cost-effectiveness ratio of implementing OxMIS was cost saving. Using probabilistic sensitivity analysis, 99.96% of 10,000 simulations remained cost saving.

**Conclusion:**

Cost-effectiveness analysis can be conducted on risk prediction models. Implementing one such model that focuses on suicide risk in a high-risk population can lead to cost savings and improved health outcomes, especially if explicitly linked to preventative treatments.

## Introduction

People with severe mental illnesses (SMI), defined as either schizophrenia-spectrum disorders or bipolar disorder, have increased risks of suicide that are estimated to be increased up to 20-fold more than the general population [1, 2], a relative risk higher than most other psychiatric disorders. Assessing the risk of suicide and linking this assessment to preventative measures is a central component of clinical care [3]. In some clinical settings, a small proportion of high-risk patients can account for a large proportion of available healthcare resources and costs due to repeat self-harm and near-lethal suicide attempts [4]. Interventions including medication, psychological therapies, and harm reduction measures, alone or in combination, can reduce suicidal behavior in high-risk patients [5]. Antipsychotic medication, and in particular clozapine, has been shown to reduce suicidal behavior in people with schizophrenia [6], including suicide mortality in observational studies [7].

Identifying those most at risk, and implementing effective interventions, may help improve outcomes for high-risk patient groups, reduce emergency care, and direct resources to those who can benefit the most. However, clinicians are not accurate in estimating suicide risk when relying on clinical judgment alone. Around 9 out of 10 people who died by suicide were rated at a low or no immediate risk of suicide before their death by health professionals in a national survey of UK psychiatrists [8]. Furthermore, there is other evidence to show that clinicians are overly optimistic in their risk judgments, and weigh recent factors too heavily [9, 10]. Despite the centrality of suicide assessment and prevention in clinical care, previous research has identified limitations in current approaches [11], which predominantly draw on tools and checklists developed for other purposes, not developed using multivariable models, and tested for outcomes apart from suicide mortality (such as suicidal ideas or self-harm) [12, 13].

National clinical guidelines recommend risk assessment for people at risk of self-harm with SMI [14, 15]. Specifically, they state that risk assessments should take the form of a comprehensive psychosocial assessment to include needs, risk and protective factors, and the identification of safety concerns. In England, these guidelines do not recommend any current tools. In order to improve risk assessment, high-quality research methods developing and validating tools, and their linkage to preventative methods needs to be conducted. Risk prediction tools can provide structure and consistency to risk assessment, and inform and challenge *a priori* assumptions. Using risk prediction tools in combination with clinical judgment may improve care by identifying those at higher risk earlier and lead to more targeted management. Those identified correctly as low-risk can avoid unnecessary further assessments and interventions, with a reduction of associated adverse outcomes and additional healthcare costs. In addition, structured risk assessment provides an opportunity to involve patients and families in clinical decision-making, providing a more objective framework to aid discussions within the multidisciplinary mental health team. Importantly, a survey of clinicians with patients who died by suicide found that the clinicians thought that suicide risk assessment tools could be a helpful adjunct, although there were concerns about lack of training and user-friendliness [13]. To the best of our knowledge, no previous research exists on the cost-effectiveness of suicide risk prediction tools. However, one recent study found that with sufficient accuracy such tools would provide good cost-effectiveness in the context of the US, with several pre-existing tools exceeding the necessary accuracy levels [16].

Oxford Mental Illness and Suicide (OxMIS) is a suicide risk prediction tool [17]. To the best of our knowledge, it is the first suicide risk prediction tool to be developed specifically for people with SMI. It provides a probabilistic estimate for the risk of suicide in people with schizophrenia-spectrum disorders and bipolar disorder in the 12 months after assessment, and can be used as a complement to individual needs-based risk assessment. The tool also provides a high- and low-risk categorization for research purposes, based on a pre-specified 1% suicide risk cut-off. In external validation, the tool performed well on calibration (how closely the predicted and observed probabilities are associated) and moderately well on discrimination, with a c-index of 0.71—the latter of which assumes categorical classifications of suicide risk (i.e., based on a threshold to categorize people into risk categories). As a new risk assessment tool, in addition to face validity, OxMIS requires a range of information to support its adoption into clinical practice. Previous research [18] has shown that OxMIS can feasibly be calculated from routinely collected electronic health records. However, an estimate of its potential value for money needs to be provided to guide service development and funding. Economic evaluation can provide relevant information as it compares alternative courses of action in terms of costs and effectiveness. The aim of this study was to undertake early economic modeling to estimate the potential cost-effectiveness of OxMIS compared with care as usual (i.e., no structured suicide risk assessment tool) provided to patients with SMI in secondary mental healthcare services over a 1-year time horizon. This is a vital period after patients first present to the hospital with nonfatal self-harm, as suicide rates are considerably higher in the first year after presentation than in the proceeding years [19]. Furthermore, it will provide insight into whether additional evidence should be developed to explore the long-term cost-effectiveness of OxMIS.

## Methods

### Decision analytic approach

We developed a decision analytic model to estimate the potential cost-effectiveness of using OxMIS as part of clinical decision-making in secondary care. The comparator was usual care, which for the purpose of our model includes clinical assessment of suicide risk without the use of OxMIS or any other structured risk assessment model for suicide. Cost-effectiveness was measured in terms of costs per quality-adjusted years (QALYs) gained over a year time horizon. Costs were reported in 2021 UK Pounds taking the NHS perspective as recommended in NICE guidelines [20]. The methods and results were reported following the Consolidated Health Economic Evaluation Reporting Standards (CHEERS) guidelines [21].

### Model structure

Patients with SMI enter the decision tree on their first inpatient or outpatient visit to secondary mental health care. Individuals may then be assessed for their risk of suicide either using OxMIS independently of clinical judgment or using clinical judgment alone. Those assessed to be at elevated risk receive what we have termed “high-risk management” (HRM), and conversely, those at low-risk receive “low-risk management” (LRM). In this model, HRM is a treatment plan to reduce suicide. In practice, the components of HRM may include any or all of the following interventions: medication review, increased frequency of clinical reviews, multidisciplinary assessment, elective admission, or emergency admission. We assumed that HRM comprised four additional clinical reviews and a multidisciplinary psychosocial assessment. Following assignment to a management plan, patients may die by suicide or continue to remain in clinical care. Death by other causes was not included in the model as it would be equal with and without OxMIS.

**Figure 1.**
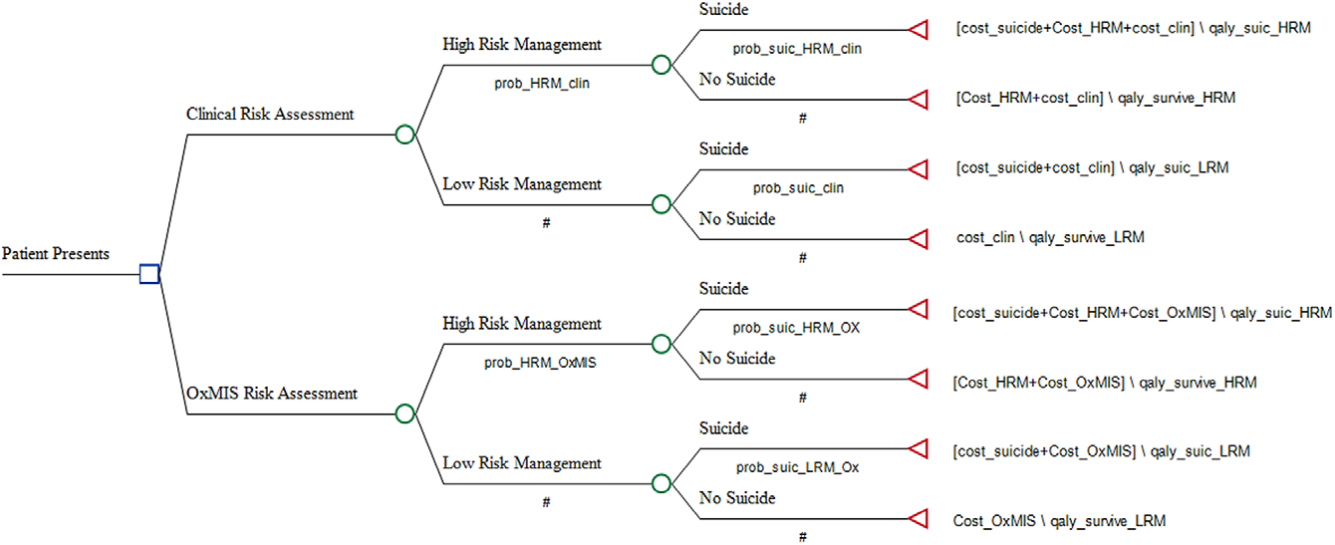
Decision tree modeling the suicide outcomes and costs following risk assessment with OxMIS or unstructured clinical assessment.

A decision tree was constructed to represent the journey of patients with SMI presenting for risk assessment in secondary care (Figure 1).

### Model parameters

OxMIS’s true negative rate (or specificity) was 75% in external validation, which is the basis on which we have assigned an elevated risk to 25% [17]. For clinical assessment alone, we used a probability of 0.5 for the chance of being assessed as needing HRM or LRM, based on the low sensitivity and specificity of clinical risk assessment [8]. We have assumed that without a structured suicide risk assessment tool, clinicians assess half of the patients as higher risk of suicide and requiring HRM. Conversely, half of the patients seen will be assessed as being at a low risk of suicide and will be managed conservatively. The 50% chance of receiving LRM or HRM was an assumption that half the people who have a SMI have a HRM plan (in addition to usual care), and the others receive usual care without these additional elements. We think that this is broadly consistent with a real-world psychiatric service, where the number of referrals and follow-up appointments would not lend itself to HRM of all patients. The effects on suicide risk following HRM were based primarily on trial evidence such as RCTs showing reduced odds of suicide in SMI patients taking lithium and antipsychotics [7, 22] but other treatments were considered. Given the wide variety of management practices to reduce self-harm and suicide in high-risk patients, we applied a conservative estimate of a 10% reduction of the risk of suicide following HRM. The risk of suicide following a higher OxMIS score was 0.017 (or 1.7%), derived from the number of people correctly identified by OxMIS to subsequently die by suicide within 12 months. The risk of suicide in the treatment-as-usual arm was a 12-month incidence of suicide in the SMI population of 0.008 (or 0.8%) [17].

The cost associated with completing a risk assessment using OxMIS during an outpatient mental health clinic was estimated to be £57, assuming an additional 10 min (i.e., 1/6th) of an outpatient review in adult general psychiatry, using 2019/20 NHS Reference Costs [23] (last updated in April 2022). Conducting a risk assessment without OxMIS was set to £253 based on the estimated cost of psychosocial assessment by Tsiachristas 2017 inflated to 2021 values [24], as almost all SMI patients are managed as outpatients in England. A cost of £1,615 was estimated for HRM, comprising of an additional four clinical reviews at £343 each (2019/20 NHS Reference Costs for an outpatient review in adult general psychiatry) [23] and a multidisciplinary psychosocial assessment at £253 [24]. The costs of medication and associated monitoring were not added to this figure, but were assumed to be included in the cost of additional reviews. Conversely, LRM was assumed to involve no additional suicide risk management, and incur no additional costs. The cost of suicide was obtained from Public Health England’s Mental Health Promotion Return on Investment tool [25], and its supporting evidence [26]. We used the direct cost of suicide associated with immediate emergency service and health-related costs. The cost was £260 at 2022 prices (based on £227 at 2015 prices).

The baseline weighted utility for living with SMI was 0.77 (where 0 is death and 1 is maximum health), based on previous cost-effectiveness studies of this population [27, 28]. Treatment during HRM was associated with a reduction of utility of 0.05, based on a previous study of the effect of antipsychotic treatment on public preferences for health states [29]. We assigned a utility for the state of death by suicide as 0 [30].

The parameters used in the model are shown in Table 1.

**Table 1.** Probabilities, costs, and QALYs used to develop a base case scenario modeling the use of OxMIS in secondary care.

Input parameter	Deterministic value (as a proportion)	Distribution in probabilistic sensitivity analysis	Distribution parameters	Source
Probability of OxMIS score indicating higher risk	0.25	Beta	Alpha = 74.75 Beta = 224.25	Fazel et al. [17]
Probability of clinical assessment indicating high-risk	0.5	Beta	Alpha = 49.50 Beta = 49.50	Fazel et al. [17]
Reduction of suicide risk with treatment plan	0.10	Fixed	–	Taipale et al. [7]
Risk of suicide following higher OxMIS score	0.017	Beta	Alpha = 98.28 Beta = 5683.07	Fazel et al. [17]
Risk of suicide following clinical assessment	0.008	Beta	alpha = 99.19 beta = 12299.81	Fazel et al., [17]
Costs (£)				
Clinical assessment	253	Gamma	Alpha = 2.78 Lambda = 0.01	Tsiachristas et al. [24]
OxMIS	57	Gamma	Alpha = 2.78 Lambda = 0.05	NHS England [23]
High-risk management	1,615	Gamma	Alpha = 2.78 Lambda = 0.001	NHS England [23] and Tsiachristas et al. [24]
Suicide	260	Gamma	Alpha = 2.78 Lambda = 0.01	PHE Return on Investment Tool [25]
QALYs				
Survived after low-risk management	0.77	Normal	SD = 0.08	Dilla et al. [27]
Survived after high-risk management	0.73	Normal	SD = 0.07	Lenert et al. [29]
Suicide after low-risk management	0.385	Normal	SD = 0.04	Assumption based on Dilla et al. [27]
Suicide after high-risk management	0.365	Normal	SD = 0.03	Assumption based on Lenert et al. [27]

*Notes*: QALYs of survivors were estimated based on the assumption that they had the same quality of life (i.e., utilities) throughout the 1-year time horizon. For those who died by suicide, QALYs were halved assuming that suicide was completed at midpoint (i.e., 6 months after the assessment).

Abbreviations: OxMIS, Oxford Mental Illness and Suicide; QALYs, quality-adjusted life years; SD, standard deviation.

### Scenario analysis

We constructed a scenario whereby OxMIS was used following clinical judgment, rather than independently. For this analysis, we hypothesized that undertaking OxMIS following a clinical assessment alters the post-test probability. The change between the pre- and post-test probability can be estimated using likelihood ratios, which are in turn derived from the sensitivity and specificity of the test [31]. This estimates the change in the probability of correctly identifying or excluding an outcome of following the administering a test. Using likelihood ratios, OxMIS changes the probability of a clinician assessment as higher risk being a true positive (whereby the patient is correctly identified to die by suicide) from 0.5 to 0.69. This change in probability was used to adjust the probability of patients entering HRM and LRM following risk assessment with clinical judgment followed by OxMIS.

### Sensitivity analysis

We assessed the robustness of the underlying parameters and assumptions through univariate sensitivity analysis, and multivariable probabilistic sensitivity analysis using Monte Carlo simulation. Using the base case analysis, we modeled the uncertainty associated with chosen parameters (cost of OxMIS, cost of HRM, cost of suicide, utility of HRM, probability of being assessed as higher risk following the use of OxMIS, and probability of suicide given a risk score from OxMIS of over 5%) by randomly drawing a value from within their distributions and using it as input parameter in the model simultaneously and then calculating incremental cost-effectiveness. This process was repeated 10,000 times. The results of all 10,000 combinations of estimated incremental costs and incremental QALYs were displayed visually on a cost-effectiveness plane (Table 2).

**Table 2.** Incremental cost-effectiveness ratios, costs and QALYs with and without OxMIS suicide risk assessments.

	Assessment with OxMIS Mean (SD)	Assessment without OxMIS Mean (SD)	Difference Mean (95% CI)
Main analysis			
Cost	£463 (245)	£1,062 (508)	−£599 (−1,321;-156)
QALYs	0.76 (0.06)	0.75 (0.05)	0.01 (−0.04;0.07)
ICER	Dominant		
Scenario analysis			
Cost	£813 (337)	£1,062 (508)	−£250 (−786;31)
QALYs	0.75 (0.06)	0.75 (0.05)	0.01 (−0.03;0.05)
ICER	Dominant		

Abbreviations: ICER, incremental cost-effectiveness ratio; OxMIS, Oxford Mental Illness and Suicide; QALYs, quality-adjusted life years; SD, standard deviation.

## Results

### Cost-effectiveness analysis

In our model, conducting suicide risk assessment with OxMIS was potentially cheaper and associated with a small increase in quality of life. In our main analysis, mean costs for assessment with OxMIS were £463 compared to £1,062 without OxMIS. There was a small increase (0.01) in QALYs after an OxMIS assessment. In both the main and scenario analysis, OxMIS was dominant (i.e., cheaper and associated with an increase in quality of life).

### Sensitivity analyses

**Figure 2.**
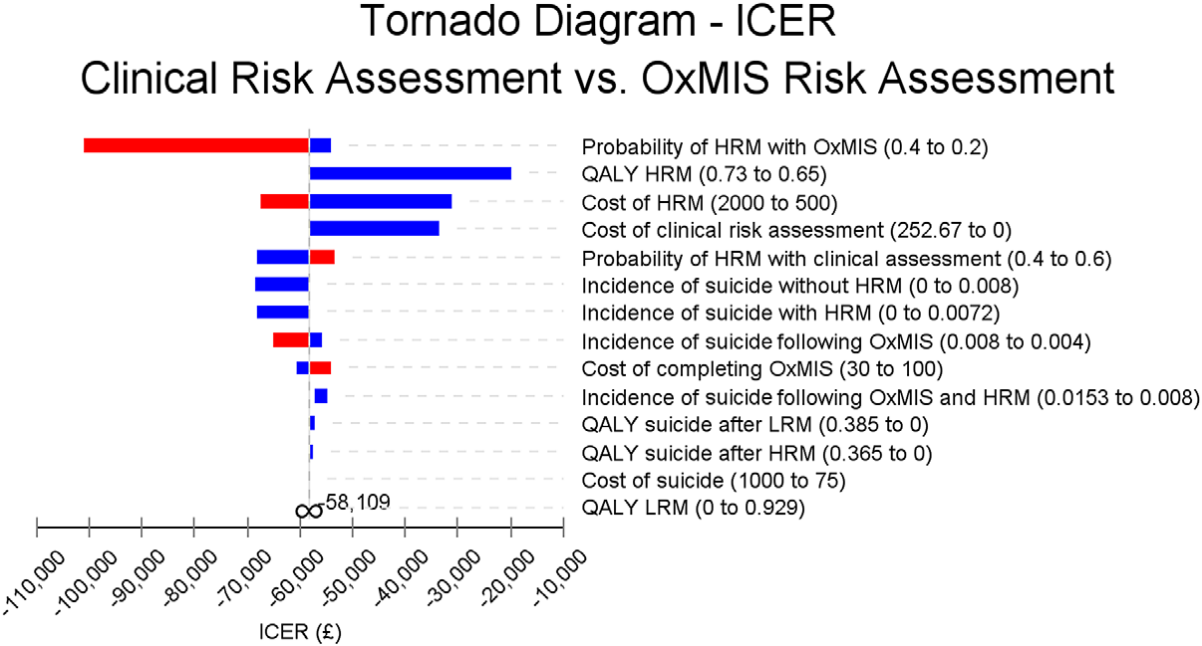
Impact of univariable sensitivity analyses on model uncertainty estimating incremental cost-effectiveness ratios (ICERs). HRM, high-risk management; LRM, low-risk management. Vertical line represents the mean ICER of −£58,109 from the base case scenario. Red bar segments indicate that the value of each parameter has increased, while blue segments show parameter values have fallen. Values to the right of the vertical base case scenario line indicate less favorable cost-effectiveness with the cost per QALY increasing compared to the base case scenario, while those to the left indicate an improvement in cost-effectiveness, with an increased saving per QALY gained.

The univariable sensitivity analysis showed that OxMIS was still a dominant alternative after varying individual parameters in the model maintained the cost-effectiveness of risk assessment by OxMIS (Figure 2). Incremental cost-effectiveness ratios (ICERs; the incremental cost for each additional QALY gained) ranged from −£101,036 to −£19,769 per QALY (representing savings per QALY). Probability of HRM with OxMIS had the biggest influence on cost-effectiveness. Conversely, the cost of suicide had the smallest influence on cost-effectiveness.

**Figure 3.**
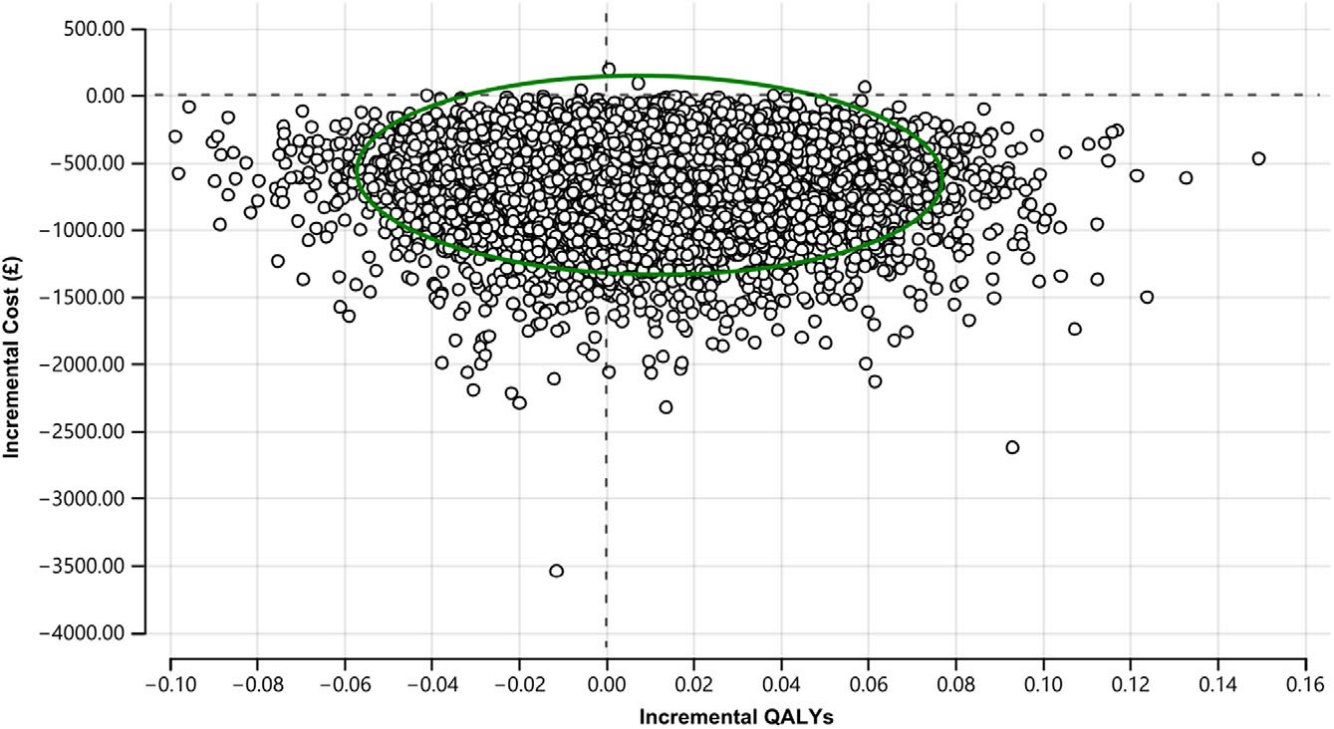
Cost-effectiveness plane of main analysis (10,000 simulated ICERs). The green circle represents the 95% confidence ellipse.

The uncertainty around the estimated ICER is presented in Figure 3, where all 10,000 simulated ICERs are plotted. A total of 99.96% of all simulations were plotted in the low half of the cost-effectiveness plane, meaning high certainty in the estimated cost savings. In 61% of the 10,000 simulations, adding OxMIS on top of clinical assessment was dominant (i.e., cheaper and more effective) than clinical assessment alone. However, in 35% of the simulated ICERs, OxMIS alone was cheaper but less effective than clinical assessment. There was a very low likelihood (i.e., 2.4%) that the addition of OxMIS risk assessment to clinical practice would be inferior (i.e., more expensive and less effective) than clinical assessment alone.

## Discussion

In this study, we developed an economic model to investigate the potential outcome of implementing an evidence-based suicide risk prediction tool (OxMIS) into secondary mental health care. The economic model combined data from previous cohort, trial, economic studies, the development study of OxMIS, and the prevalence of suicides in patients with SMI (schizophrenia-spectrum disorders and bipolar disorder). It was thus dependent on the validity of existing data, which was tested in sensitivity analyses. The economic model determined the potential cost-effectiveness of using OxMIS to identify those more likely to die by suicide and link this identification to preventative interventions. Costs included health and immediate emergency care service costs, and effectiveness was measured in terms of QALYs.

Our study suggests that using OxMIS during suicide risk assessment for patients with SMI is associated with a mean cost-saving of around £300 per assessed patient and is associated with a marginal gain in 0.01 QALYs per year. Such findings support the case for developing and identifying further evidence on the long-term costs and benefits of OxMIS, in order to estimate cost-effectiveness over a lifetime horizon. Although the associated improvements in effectiveness as measured by QALYs were small, there was a reduction in the number of people exposed to the harms associated with suicide prevention treatment. This is due to OxMIS accurately identifying low-risk persons, who do not require additional treatment beyond the standard provision for SMI patients. In our model, these harms are a consequence of the side effects of medications, particularly clozapine [29]. These findings were consistent across different models and remained during sensitivity analyses that varied the values of our input parameters either in isolation or combination. The high negative predictive value of OxMIS (which is reported to be 99%) contributes to the identification of these low-risk people. This is particularly relevant in suicide prevention because the incidence of suicide is low, and so the majority of people receiving preventative treatment would not have otherwise died by suicide. The cost of administering OxMIS in clinical settings will also have a bearing on its cost-effectiveness, and so approaches to enable clinicians to quickly and easily use the tool would be beneficial. Previous research has shown that OxMIS could be calculated from routinely collected data in electronic health records [18], and as it is based on 17 easy-to-score items, this suggests that the tool is scalable.

Overall, the results contribute to existing evidence suggesting the economic benefits of suicide prevention measures [26] and the cost-effectiveness of psychosocial assessment in preventing repeat self-harm [32]. We did not identify any previous research has been previously conducted on the cost-effectiveness of suicide risk prediction tools. Such tools may have synergetic effects when combined with other secondary care suicide prevention measures that have been found to be cost-effective. For example, universal screening by nurses of emergency department patients, alongside a 12-month telephone intervention, was deemed cost-effective when compared to universal screening alone and treatment-as-usual [33]. Similarly, sending follow-up postcards to hospital emergency department patients was found to be cost-effective, as was providing an additional telephone outreach focused on cognitive behavioral therapy [34].

In addition, we have provided an approach to model the cost-effectiveness of risk prediction tools, which have been increasing in number and complexity in mental health [12]. The main elements that researchers should consider are drawing on different research designs to provide data on model parameters, and a range of sensitivity analyses to test model assumptions. As such assumptions are inevitable due to the lack of research in all areas relevant to model implementation, these sensitivity analyses should be included in all cost-effectiveness studies in mental health.

### Strengths and limitations

One strength of the current study is the application of high-quality epidemiological and cost data to the models. These are based on information from the development of OxMIS, which used linked population-based registers in Sweden, and also NHS reference costs. A limitation was the model assumptions, particularly in relation to clinical assessment. There is a lack of information on how clinicians assess suicide risk without the use of structured tools, which was the basis of the assumption that half the patients with SMI would be estimated as low-risk. One related assumption was that we assumed usual care included a psychosocial assessment, whereas in clinical practice, this will vary and in some regions uptake is not high. However, probabilistic sensitivity analysis allowed for these assumptions and estimations to be tested. An additional limitation was the loss of some of the complexity of the patient journey from clinical assessment to the outcome of suicide. For example, we were unable to model the implications of suicidal attempts and self-harm that may have occurred on the pathway to suicide mortality. As a result, our model did not include the costs associated with nonfatal self-harm, which are on average £809 per episode of self-harm, with 1.4 self-harm presentations occurring per patient each year [24]. Further research on the outcomes of clinical suicide risk assessment with and without the use of structured tools would be beneficial. Costs and utilities for nonfatal suicidal events were not included in the model, as there was no available data on the risk of self-harm following risk assessment using OxMIS. Therefore, this study only looked at the effects on death by suicide. Moreover, the short time horizon of the economic evaluation may have underestimated the cost-effectiveness of OxMIS as suicide risk remains elevated for many years in high-risk persons and OxMIS treatment effect (i.e., putting patients on HRM) may last longer than a year. However, extending the time horizon would not change the reimbursement decision that is, OxMIS seems to be already cost-effective even with a short time horizon. Finally, we assumed the occurrence of a suicide to be at 6 months but further evidence is needed to test this assumption.

In summary, our results contribute to the evidence base for the implications of implementing an evidence-based risk assessment tool, such as OxMIS, into routine psychiatric care. These results should be considered alongside a particular risk assessment tool’s accuracy, face validity, acceptability, and feasibility.

## Data Availability

This economic modeling study used secondary data that are available in the literature.
